# Comparative Cytotoxicity of Commercially Pure Titanium, Silver–Palladium and Nickel–Chromium Alloys on Human Gingival Fibroblasts: An In Vitro Study

**DOI:** 10.3390/jcm15145680

**Published:** 2026-07-20

**Authors:** Passent Ellakany, Zakia AbdelRahman, Chin-Chuan Fu, Noha Abdelsamad, Akram Sayed Ahmed

**Affiliations:** 1 Division of Prosthodontics, School of Dentistry, University of Alabama at Birmingham, Birmingham, AL 35233, USAamsayeda@uab.edu (A.S.A.); 2 Department of Immunology, Medical Research Institute, Alexandria University, Alexandria 21561, Egypt; 3 Graduate Biomedical Sciences, University of Alabama at Birmingham, Birmingham, AL 35233, USA; 4 Department of Dental Biomaterials, Faculty of Dentistry, Tanta University, Tanta 31773, Egypt

**Keywords:** titanium, artificial saliva, silver-palladium, nickel-chromium alloys, human gingival fibroblast, cytotoxicity

## Abstract

**Background/Objectives:** This study aimed to evaluate the direct effect of elements released from dental casting alloys on human gingival fibroblasts (HGF). **Methods:** Ten discs each of commercially pure titanium (Ti), silver-palladium (Ag-Pd), and nickel-chromium (Ni-Cr) alloys, measuring 4 mm in diameter and 3 mm in thickness, were fabricated and placed directly on HGF cultures for 72 h. Five discs from each alloy group were incubated in DMEM, whereas the remaining five discs were incubated in artificial saliva. Cytotoxicity was assessed using three in vitro methods: morphological evaluation of HGF, the dye exclusion test (DET), and the methyl-thiazolyl-tetrazolium (MTT) colorimetric assay. **Results:** HGF directly exposed to cpTi, Ag-Pd, and Ni-Cr discs demonstrated reduced cell density around the alloy specimens compared with untreated control cells after 72 h of incubation in both DMEM and artificial saliva. cpTi alloy exhibited no detectable cytotoxic effects, whereas Ag-Pd and Ni-Cr alloys demonstrated cytotoxicity in both DMEM and artificial saliva under the experimental conditions. Among the tested alloys, Ni-Cr exhibited the highest cytotoxicity. Artificial saliva enhanced the cytotoxic effects of the tested alloys, as evidenced by increased morphological alterations, reduced cell viability, and lower MTT activity compared with DMEM. **Conclusions:** cpTi alloy exhibited the lowest direct cytotoxic effect on HGF in both DMEM and artificial saliva. In contrast, Ag-Pd and Ni-Cr alloys demonstrated greater cytotoxicity, particularly in the presence of artificial saliva, suggesting that both alloy composition and the surrounding environment influence the biological response of oral soft tissues.

## 1. Introduction

Metallic biomaterials play a fundamental role in contemporary prosthodontics and implant dentistry and are extensively used for dental implants, implant-supported prostheses, porcelain-fused-to-metal fixed dental prostheses (PFM-FDPs), removable partial denture (RPD) frameworks, and other prosthetic restorations. The long-term clinical success of these restorations depends not only on their mechanical performance but also on their biological compatibility with oral tissues [1,2,3,4].

The oral cavity represents a complex biological and electrochemical environment characterized by fluctuations in pH, temperature, microbial activity, and exposure to saliva and dietary substances. These factors may influence corrosion behavior and promote the release of metallic ions from dental alloys into the surrounding environment [5,6,7]. Released ions may interact with oral soft tissues and induce biological responses ranging from local inflammatory reactions and hypersensitivity reactions to cytotoxic and immunological effects, depending on alloy composition, ion concentration, and duration of exposure [8,9,10,11].

Corrosion resistance and biocompatibility are therefore critical considerations when selecting metallic materials for dental applications. Corrosion resistance and biocompatibility are fundamental criteria in the selection of metallic dental biomaterials because corrosion-induced degradation may compromise both the longevity of prosthetic restorations and the biological response of surrounding tissues. The release of metallic ions resulting from electrochemical corrosion may alter cellular metabolism, induce oxidative stress, stimulate inflammatory pathways, and trigger hypersensitivity reactions, particularly in susceptible individuals. Consequently, long-term clinical success depends not only on the mechanical performance of dental alloys but also on their ability to maintain chemical stability and minimize ion release under the complex conditions of the oral environment [12,13,14].

Commercially pure titanium (cpTi) and titanium-based alloys are considered the gold standard for dental implant applications because of their excellent corrosion resistance, favorable tissue response, and ability to form a stable titanium oxide layer that limits ion release and promotes osseointegration [15,16,17]. Recent investigations continue to support the favorable biological performance of titanium-based materials while emphasizing the importance of understanding metal ion release, tribocorrosion, and tissue interactions at implant-abutment interfaces [18,19].

Base-metal alloys, including nickel-chromium (Ni-Cr) and cobalt-chromium (Co-Cr), remain widely used in fixed and removable prosthodontics because of their favorable mechanical properties, durability, and relatively low cost [8,20,21]. However, concerns regarding the biological effects of released nickel and chromium ions persist because these elements have been associated with cytotoxicity, hypersensitivity reactions, inflammatory responses, and alterations in cellular metabolism [10,22,23,24]. Recent studies have demonstrated that alloy composition, manufacturing techniques, and environmental conditions significantly influence corrosion behavior, ion release, and biocompatibility [8,13,24].

Nickel and chromium ions exhibit distinct biological effects after being released from dental alloys. Nickel is recognized as one of the most common metal sensitizers and has been associated with allergic contact reactions, oxidative stress, mitochondrial dysfunction, apoptosis, and reduced fibroblast viability. Chromium ions, particularly when released under corrosive conditions, may further enhance inflammatory responses through increased production of reactive oxygen species and pro-inflammatory cytokines. The combined release of nickel and chromium may therefore adversely influence peri-prosthetic soft tissue healing and compromise long-term biocompatibility [12,14,25].

Manufacturing methods, including conventional casting, subtractive machining, and additive manufacturing, may alter alloy microstructure, grain size, surface roughness, residual stresses, and oxide layer formation, thereby influencing corrosion resistance, metallic ion release, and subsequent biological response. Surface defects, residual porosity, microstructural heterogeneity, and post-processing procedures such as polishing and heat treatment affect passive oxide layer formation, corrosion resistance, and subsequent metal ion release. Recent investigations have demonstrated that improved finishing and polishing procedures significantly reduce surface roughness and ion release while enhancing corrosion resistance and overall biocompatibility of dental alloys [12,13,26].

Although the mechanical properties of dental alloys have been extensively investigated, their biological interactions with oral tissues remain an important area of research. Human gingival fibroblasts (HGF) were selected because they are among the primary cell populations exposed to restorative materials and released ions in the oral cavity and provide a clinically relevant model for evaluating the biocompatibility, cytotoxicity, and inflammatory responses associated with dental biomaterials [17,27,28,29].

Artificial saliva has been widely used as an experimental medium to simulate oral conditions during the evaluation of dental biomaterials [13,30]. However, the biological response of oral soft tissues to ions released from commonly used prosthodontic alloys under different environmental conditions remains incompletely understood. Commercially pure titanium (cpTi), silver–palladium (Ag-Pd), and nickel–chromium (Ni-Cr) alloys were selected because they represent three of the most widely used metallic biomaterials in prosthodontics with distinctly different compositions, corrosion behavior, and biological characteristics. Commercially pure titanium is considered the gold standard because of its excellent corrosion resistance and biocompatibility, whereas Ag-Pd alloys are widely used noble alloys with intermediate corrosion resistance. In contrast, Ni-Cr alloys are economical base-metal alloys that have been associated with greater metallic ion release and increased cytotoxic potential. Direct comparison of these clinically relevant alloys under identical experimental conditions is limited [17,29,30,31]. This comparison provides a better understanding of how alloy composition influences the biological response of human gingival fibroblasts and may assist clinicians in selecting biomaterials with favorable biological performance. Therefore, further investigation is needed to clarify the influence of alloy composition and environmental conditions on cellular response and biocompatibility.

The present study aimed to evaluate the cytotoxic effects of commercially pure titanium (cpTi), silver-palladium (Ag-Pd), and nickel-chromium (Ni-Cr) alloys on human gingival fibroblasts using morphological assessment, the dye exclusion test (DET), and the methyl-thiazolyl-tetrazolium (MTT) assay. In addition, the influence of artificial saliva and cell culture medium on the biological behavior of these commonly used dental alloys was investigated. The null hypothesis states that no significant cytotoxic effect would be noticed between the different tested dental alloys.

## 2. Materials and Methods

### 2.1. Study Design and Sample Size

**Figure 1:**
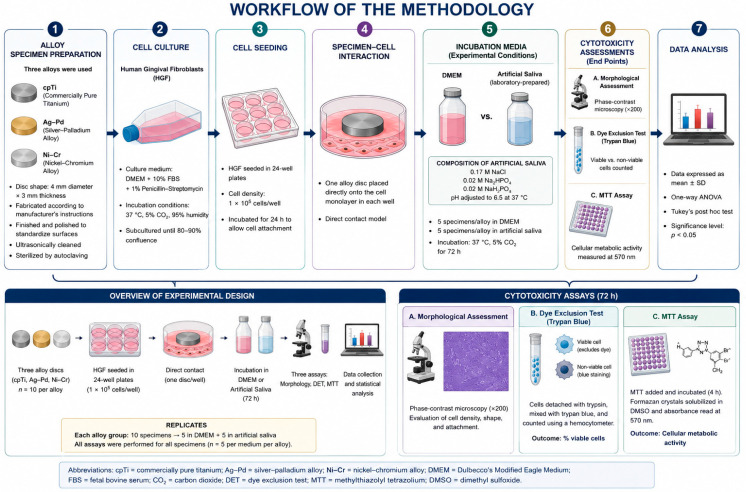
Workflow of the methodology.

The present in vitro study evaluated the cytotoxic effects of three dental casting alloys—commercially pure titanium (cpTi), silver-palladium (Ag-Pd), and nickel-chromium (Ni-Cr)—on human gingival fibroblasts (HGF) (Table 1). The sample size was determined based on previous in vitro studies evaluating the cytotoxicity of dental alloys and biomaterials using similar experimental designs [22,32,33]. Ten specimens were included for each alloy group to ensure adequate representation and to compensate for potential laboratory processing errors. Therefore, the total sample consisted of 30 specimens (*n* = 10 per alloy group, Figure 1).

### 2.2. Alloy Specimen Preparation

Commercially pure titanium (cpTi; ASTM Grade 2, Nippon Steel Corporation, Tokyo, Japan) bars were sectioned using an Elite BB25-1 lathe milling machine to produce disc-shaped specimens measuring 4 mm in diameter and 3 mm in thickness. The specimens were subsequently polished and finished according to the manufacturer’s recommendations.

Silver–palladium (Ag-Pd; IPS d.SIGN^®^ 53, Ivoclar Vivadent, Schaan, Liechtenstein) and nickel–chromium (Ni-Cr; IPS d.SIGN^®^ 15, Ivoclar Vivadent, Schaan, Liechtenstein) specimens were fabricated using the conventional lost-wax casting technique. Standardized wax patterns were produced using a custom-made split mold to obtain disc-shaped specimens measuring 4 mm in diameter and 3 mm in thickness. Following divestment, all cast specimens underwent standardized laboratory finishing and polishing procedures according to the manufacturer’s recommendations to produce smooth, standardized surfaces before sterilization and biological evaluation.

### 2.3. Incubation Media

To evaluate the influence of environmental conditions on alloy cytotoxicity, specimens from each alloy group were randomly divided into two subgroups (*n* = 5). One subgroup was incubated in Dulbecco’s Modified Eagle Medium (DMEM) to simulate conditions in areas with limited saliva exposure, such as the gingival sulcus. The second subgroup was incubated in artificial saliva to simulate oral conditions in which prosthetic materials are directly exposed to saliva [11,29]. The artificial saliva consisted of 0.17 mol/L sodium chloride (NaCl), 0.02 mol/L disodium hydrogen phosphate (Na_2_HPO_4_), and 0.02 mol/L sodium dihydrogen phosphate (NaH_2_PO_4_), adjusted to pH 6.5 at 37 °C. The solution was prepared in the laboratory using analytical-grade reagents (Sigma-Aldrich, St. Louis, MO, USA) and was not commercially obtained.

DMEM was selected to simulate peri-prosthetic connective tissue conditions where restorative materials remain in contact primarily with gingival fibroblasts and tissue fluids, such as the peri-implant sulcus or subgingival margins of fixed prostheses. In contrast, artificial saliva was used to reproduce the electrochemical oral environment surrounding restorations directly exposed to saliva, where electrolyte composition and pH may influence corrosion behavior and metallic ion release. Evaluating both media, therefore, provides a more comprehensive assessment of alloy biocompatibility under clinically relevant conditions [12,13,14].

### 2.4. Sterilization Procedure

Prior to testing, all specimens were cleaned using Alconox detergent and a soft brush, followed by rinsing with distilled water. The discs were then ultrasonically cleaned in 70% isopropyl alcohol (Sigma-Aldrich, St. Louis, MO, USA) for 5 min. After removal of alcohol residues using sterile distilled water, specimens were dried in sterile culture plates at 60 °C for 48 h before experimentation.

### 2.5. Human Gingival Fibroblast Culture

The study protocol was approved by the Institutional Review Board of the Medical Research Institute (IRB No. 2019052), and informed consent was obtained from all participants.

Human gingival fibroblasts were isolated from healthy gingival tissues obtained during routine premolar extraction procedures and cultured according to previously published protocols [22,28,29]. Tissue specimens were cultured in DMEM supplemented with 15% fetal bovine serum (FBS), 100 U/mL penicillin G, 100 μg/mL streptomycin, and 50 ng/mL amphotericin B (Gibco, Thermo Fisher Scientific, Waltham, MA, USA). Gingival tissues were sectioned into small fragments and transferred into tissue culture flasks containing complete culture medium.

Cells were incubated at 37 °C in a humidified atmosphere containing 5% CO_2_. After reaching approximately 75% confluence, cells were detached using 0.05% trypsin and 0.02% EDTA and subcultured. Culture medium was replaced twice weekly. Cell viability was assessed using 0.2% trypan blue exclusion, and only cultures demonstrating greater than 90% viability were included in the experiments.

### 2.6. Morphological Assessment Method

A total of 12,500 HGF cells were seeded into each well of a 24-well tissue culture plate (Costar-Corning, Sigma-Aldrich). Alloy discs were immediately placed in direct contact with the cultured cells. Cellular responses were evaluated in either DMEM or artificial saliva (0.17 mol/L NaCl, 0.02 mol/L Na_2_HPO_4_, 0.02 mol/L NaH_2_PO_4_; pH 6.5).

Untreated wells served as negative controls, while formalin-treated cultures served as positive controls. Teflon discs of identical dimensions were used as material controls.

After 72 h of incubation, cellular morphology was evaluated using an inverted light microscope by a blinded examiner. The area of cell lysis surrounding each specimen was scored according to previously published criteria [23,32]. The percentage of apoptotic cells was also assessed microscopically and scored as follows: 0 (<10%), 1 (10–20%), 2 (20–40%), and 3 (40–60%).

A 72-h incubation period was selected because it is widely used in in vitro cytotoxicity studies and provides sufficient time for cellular attachment, early proliferation, and the biological effects of released alloy components to become detectable. This incubation period has been adopted in previous investigations evaluating the cytotoxicity of dental metallic biomaterials and complies with commonly accepted approaches for in vitro biocompatibility assessment.

Median scores were calculated for each specimen. Cytotoxicity was classified as non-cytotoxic (0–0.5), mildly cytotoxic (0.6–1.9), moderately cytotoxic (2.0–3.9), or severely cytotoxic (4.0–5.0), according to previously published cytotoxicity assessment criteria [33,34].

### 2.7. Dye Exclusion Test (DET)

Cell viability was assessed using the trypan blue dye exclusion test. Viability was calculated as the percentage of viable cells relative to the total cell count. Relative viability was expressed as a percentage of the negative control group.

### 2.8. MTT Cytotoxicity Assay

Cellular metabolic activity was evaluated using the methyl-thiazolyl-tetrazolium (MTT) assay. This assay is based on the reduction of tetrazolium salt into insoluble formazan crystals by mitochondrial succinate dehydrogenase enzymes in viable cells. The amount of formazan produced is directly proportional to the number of metabolically active cells [35].

### 2.9. Statistical Analysis

Statistical analyses were performed using IBM SPSS Statistics software (Version 23.0; IBM Corp., Armonk, NY, USA). Descriptive statistics were calculated as means and standard deviations. Differences among groups were analyzed using one-way analysis of variance (ANOVA). Statistical significance was established at *p* < 0.05.

## 3. Results

### 3.1. Morphological Assessment

**Figure 2:**
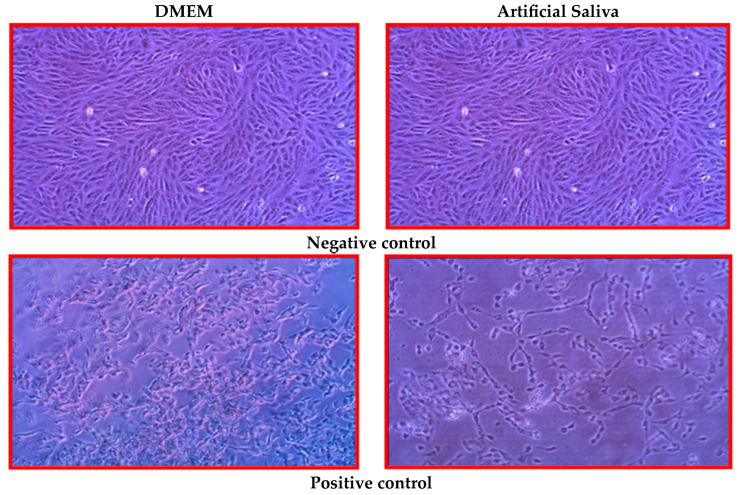
Demonstrate HGF in negative & positive controls in both DMEM media and artificial saliva. (Original magnification ×200).

**Figure 3:**
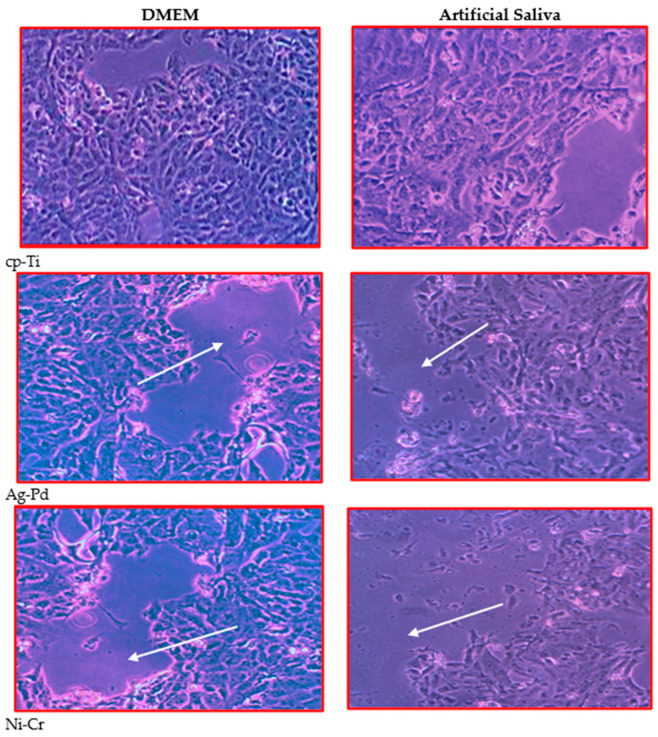
HGF in direct contact with different alloys in both DMEM media and artificial saliva. (White arrow indicates dead area under the alloy) (Original magnification ×200).

The morphological response of human gingival fibroblasts (HGF) following direct exposure to commercially pure titanium (cpTi), silver–palladium (Ag-Pd), and nickel–chromium (Ni-Cr) alloys for 72 h is presented in Table 2 and illustrated in Figure 2 and Figure 3. Untreated cells (negative control) exhibited the typical spindle-shaped fibroblastic morphology with homogeneous distribution, normal cell attachment, and high cell density throughout the culture wells.

Commercially pure titanium produced only minimal morphological alterations under both incubation conditions. The median cytotoxicity score remained 0.5 in both DMEM and artificial saliva, corresponding to non-cytotoxic behavior. Cells retained their characteristic spindle-shaped morphology with only slight reductions in cell density adjacent to the alloy discs and no evident increase in cellular lysis.

In contrast, Ag-Pd alloy induced localized reductions in cell density together with mild morphological alterations, including partial loss of cell attachment and limited areas of cellular lysis. Median cytotoxicity scores increased from 1.0 in DMEM to 1.5 in artificial saliva, indicating mild cytotoxicity, with more pronounced cellular alterations observed in artificial saliva.

Nickel–chromium alloy demonstrated the greatest morphological changes among the investigated materials. Cells exposed to Ni-Cr exhibited extensive areas of cellular lysis, marked reductions in cell density, increased numbers of rounded and detached cells, and loss of normal fibroblastic morphology. Median cytotoxicity scores increased from 2.5 in DMEM to 3.0 in artificial saliva, corresponding to moderate cytotoxicity.

Overall, all alloys demonstrated more pronounced morphological alterations following incubation in artificial saliva than in DMEM, suggesting that the simulated oral electrolyte environment enhanced the cytotoxic response under the present experimental conditions.

### 3.2. Cell Viability Assessment (Dye Exclusion Test)

The results of the trypan blue dye exclusion test are summarized in Table 3. Cell viability was expressed as the percentage of viable HGF relative to negative control after 72 h of direct exposure to the investigated alloys.

cpTi demonstrated the highest cell viability (90.2 ± 0.51% in DMEM and 86.3 ± 0.99% in artificial saliva), whereas Ni-Cr demonstrated the lowest viability (67.5 ± 1.54% and 54.1 ± 2.07%, respectively). The percentage of viable cells remained comparable to that of the negative control in both incubation media, indicating minimal adverse effects on HGF survival. Exposure to Ag-Pd resulted in a reduction in the percentage of viable cells compared with the control group in both DMEM and artificial saliva. However, the decrease in viability was less pronounced than that observed for Ni-Cr.

Nickel–chromium alloy exhibited the lowest HGF viability among the three investigated materials. Cell viability was markedly reduced following exposure to Ni-Cr in both incubation media, indicating a greater detrimental effect on fibroblast survival than either cpTi or Ag-Pd.

Comparison of the incubation media demonstrated a consistent trend across all alloy groups. Cell viability was lower following incubation in artificial saliva than in DMEM, suggesting that the simulated oral electrolyte environment enhanced the cytotoxic response under the present experimental conditions.

### 3.3. Cellular Metabolic Activity (MTT Cytotoxicity Assay)

The results of the MTT assay are summarized in Table 4. Cellular metabolic activity was determined by measuring mitochondrial reduction of tetrazolium salt to formazan after 72 h of direct exposure to the investigated alloys.

Commercially pure titanium demonstrated the highest metabolic activity among the materials investigated, with mean absorbance values of 0.411 ± 0.005 in DMEM and 0.383 ± 0.003 in artificial saliva, remaining close to those of the negative controls (0.458 ± 0.002 and 0.405 ± 0.003, respectively). These findings indicate preservation of mitochondrial function and minimal cytotoxicity.

Silver–palladium alloy produced intermediate metabolic activity, with absorbance values decreasing to 0.316 ± 0.006 in DMEM and 0.294 ± 0.002 in artificial saliva, indicating moderate impairment of cellular metabolism.

Nickel–chromium alloy demonstrated the lowest metabolic activity among the tested materials, with mean absorbance values of 0.213 ± 0.003 in DMEM and 0.204 ± 0.003 in artificial saliva, representing the greatest reduction in mitochondrial activity and confirming its higher cytotoxic potential.

Across all alloy groups, cellular metabolic activity was consistently lower in artificial saliva than in DMEM, indicating that the electrolyte-rich environment enhanced the biological effects of the investigated alloys.

### 3.4. Influence of the Incubation Medium

**Figure 4:**
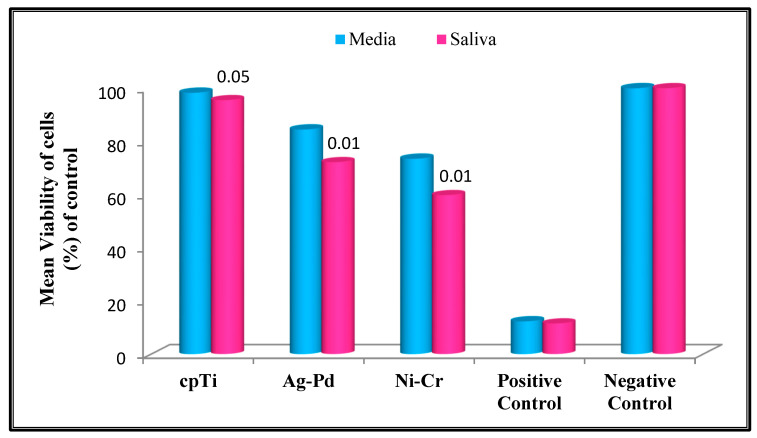
Percentage of viable HGF cells in different alloy groups. (Relative to control: 100% control).

**Figure 5:**
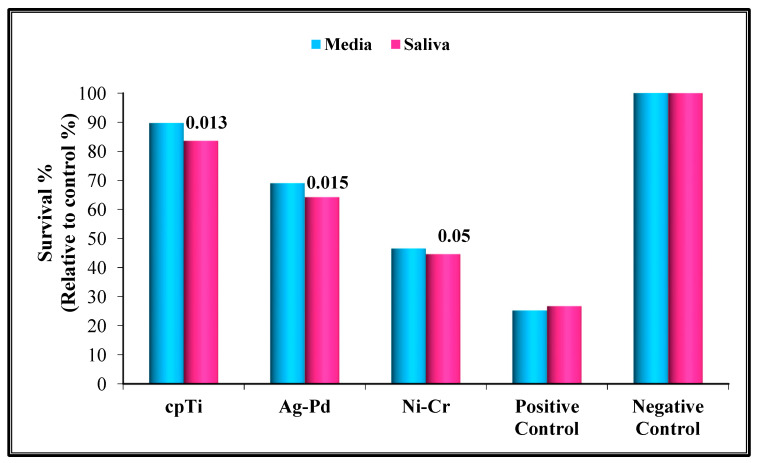
Percentage of survival (MTT) of HGF cells in different alloy groups. (Relative to control: 100% metabolic activity).

Comparison of the two-incubation media demonstrated a consistent influence of the surrounding environment on cellular behavior. Across all biological assays, specimens incubated in artificial saliva produced greater cytotoxic effects than those incubated in DMEM. This trend was reflected by larger areas of cell lysis during morphological assessment, lower percentages of viable cells in the dye exclusion test and reduced cellular metabolic activity in the MTT assay (Figure 4 and Figure 5).

Although the magnitude of these differences varied among the investigated alloys, commercially pure titanium consistently exhibited the most favorable biological response, whereas nickel–chromium demonstrated the greatest cytotoxicity under both incubation conditions. Silver–palladium showed an intermediate biological response between these two materials. The results collectively indicate that both alloy composition and incubation medium influenced the cytotoxic response of human gingival fibroblasts under the present experimental conditions.

## 4. Discussion

The biocompatibility of dental alloys is influenced by alloy composition, corrosion behavior, ion release, and the surrounding environment. Surface characteristics have an important influence on the corrosion behavior of metallic biomaterials. Increased surface roughness enlarges the effective contact area exposed to electrolytes, promotes localized corrosion, and facilitates metallic ion release, whereas polished surfaces favor the formation of stable passive oxide films that improve corrosion resistance. Recent studies have demonstrated that optimized polishing procedures reduce surface irregularities, minimize ion release, and improve the corrosion resistance of cobalt–chromium alloys, emphasizing the importance of surface finishing in maintaining long-term biocompatibility [26,36,37]. Therefore, cytotoxicity testing remains an important method for evaluating the biological safety of metallic biomaterials used in implant dentistry and prosthodontics. In the present study, artificial saliva was used to simulate the oral environment, whereas DMEM was used to represent conditions with limited direct saliva exposure, such as subgingival areas. Human gingival fibroblasts (HGF) were selected because they are among the primary cell populations exposed to restorative materials and released ions in the oral cavity and provide a clinically relevant model for evaluating the biocompatibility of dental biomaterials [17,28,29].

The MTT assay was selected because it is a well-established and sensitive method for assessing cellular metabolic activity and viability. The assay is based on the reduction of tetrazolium salts into formazan crystals by mitochondrial enzymes in viable cells, and the amount of formazan produced is directly proportional to the number of metabolically active cells [35].

The present findings demonstrated that commercially pure titanium (cpTi) exhibited the most favorable biological response among the tested alloys. No detectable cytotoxic effects were observed in either DMEM or artificial saliva. Thus, the null hypothesis was rejected. These findings are consistent with previous investigations reporting excellent biocompatibility of titanium-based materials and high survival rates of human gingival fibroblasts following exposure to titanium surfaces [17,18,19,29]. The superior biological performance of cpTi observed in the present study was reflected by the preservation of normal fibroblast morphology, the highest cell viability measured by the dye exclusion test, and the greatest metabolic activity in the MTT assay compared with Ag–Pd and Ni–Cr alloys. These findings suggest that the excellent corrosion resistance and limited ion release associated with the stable TiO_2_ passive layer effectively preserve fibroblast function under both incubation conditions. Similar observations have been reported in recent investigations evaluating titanium-based dental biomaterials, which consistently demonstrated favorable soft-tissue compatibility and high gingival fibroblast viability [15,16,18,19]. These characteristics have established titanium as the material of choice for dental implants and numerous prosthodontic applications because of its predictable biological performance and long-term clinical success.

Compared with commercially pure titanium, Ti-6Al-4V contains aluminum and vanadium as alloying elements to improve its mechanical properties. Although this alloy also develops a protective oxide layer, degradation of the passive film under corrosive conditions may result in the release of aluminum and particularly vanadium ions, which have been associated with oxidative stress, mitochondrial dysfunction, reduced cellular proliferation, and impaired fibroblast viability. Consequently, commercially pure titanium generally demonstrates superior biological performance because it combines excellent corrosion resistance with minimal ion release while avoiding the potential biological effects associated with alloying elements. Previous studies comparing cpTi and Ti-6Al-4V have consistently reported higher fibroblast viability and better cellular attachment on cpTi surfaces than on Ti-6Al-4V alloys, supporting the findings of the present investigation [17,29,38].

In contrast, Ni-Cr demonstrated the greatest cytotoxic effect on HGF among the tested alloys. This finding is consistent with previous reports indicating that nickel-containing dental alloys may exhibit increased cytotoxicity because of ion release and subsequent cellular responses [21,27]. Nickel ions have been associated with oxidative stress, inflammatory responses, hypersensitivity reactions, and alterations in mitochondrial activity, all of which may negatively affect cell viability. Chromium ions have also been associated with oxidative stress, inflammatory responses, and genotoxic effects, particularly under conditions that promote corrosion and metallic ion release from dental alloys [12,21,33]. Recent studies have further demonstrated that corrosion behavior, manufacturing techniques, alloy composition, and environmental conditions significantly influence ion release and the biological performance of base-metal alloys [8,13,24,30,33]. The higher cytotoxicity observed for Ni-Cr in the present study may therefore be attributed to increased release of nickel and chromium ions and their subsequent effects on cellular metabolism.

Although metallic ion release was not directly quantified in the present study, the greater cytotoxicity observed for Ni-Cr alloys is consistent with previous investigations reporting increased corrosion susceptibility and higher release of nickel and chromium ions under simulated oral conditions [12,13,14]. Therefore, the present findings suggest an association between the biological response observed and previously reported corrosion behavior rather than establishing a direct causal relationship.

The present study demonstrated that artificial saliva consistently enhanced the cytotoxic effects of all investigated alloys compared with DMEM. Morphological assessment revealed larger areas of cellular lysis, whereas both the dye exclusion test and MTT assay demonstrated lower cell viability and metabolic activity following incubation in artificial saliva. This finding may be associated with the electrolyte composition of artificial saliva, particularly the presence of chloride and phosphate ions, which have previously been shown to destabilize passive oxide layers, promote electrochemical corrosion, and facilitate metallic ion release from dental alloys.

Although only a single pH condition (6.5) was evaluated in the present study, previous investigations have demonstrated that acidic environments further accelerate corrosion processes, destabilize passive oxide layers, and increase metallic ion release [13,30,34,35,39]. Also, they reported that artificial saliva significantly influences corrosion behavior, surface degradation, and ion release, particularly in nickel-containing alloys, thereby adversely affecting their biological performance [13,30,34,35]. The greater reduction in fibroblast viability and metabolic activity observed in artificial saliva in the present study is therefore consistent with the greater corrosion susceptibility previously reported for dental alloys under simulated oral conditions. However, because metallic ion release was not directly quantified, these findings should be interpreted as demonstrating an association between the observed biological response and previously reported corrosion behavior rather than establishing a direct causal relationship [8,13,24].

Silver–palladium (Ag-Pd) alloys demonstrated intermediate cytotoxicity compared with commercially pure titanium and Ni-Cr alloys. This intermediate biological behavior is likely related to the alloy composition and its corrosion characteristics. Palladium is a noble metal that improves corrosion resistance by contributing to the formation of a stable passive surface layer, thereby reducing electrochemical degradation and limiting metallic ion release. However, Ag-Pd alloys are not completely inert. Under corrosive oral conditions, selective release of silver and other alloying elements may occur, which can adversely influence fibroblast viability and cellular metabolism, although to a lesser extent than Ni-Cr alloys [12,14]. The present findings are consistent with previous studies reporting that noble alloys generally exhibit greater biocompatibility than base-metal alloys because of their superior corrosion resistance and lower ion release, while remaining slightly less biocompatible than commercially pure titanium [12,14,32,33].

The present study has several limitations. First, metallic ion release was not directly quantified; therefore, correlations between ion concentration and cytotoxicity could not be established. Second, only a single evaluation period (72-h) was investigated, preventing assessment of early or long-term cellular responses. Although evaluation at additional incubation periods (e.g., 24 h) may provide further insight into the temporal progression of cytotoxicity, the present study focused on a standardized 72-h endpoint that has been widely adopted for comparative in vitro cytotoxicity assessment. Future studies should evaluate multiple time points to characterize both early and late biological responses. Third, although artificial saliva reproduced the electrolyte environment of the oral cavity, it did not fully replicate the complex composition of natural saliva, including proteins, enzymes, calcium, potassium, thiocyanate, and oral biofilm interactions. Furthermore, although all specimens underwent standardized laboratory finishing and polishing before testing, surface roughness and topography were not quantitatively measured and therefore could not be correlated with biological behavior. Future investigations should combine quantitative ion-release analysis using inductively coupled plasma mass spectrometry (ICP-MS), surface characterization techniques, multiple incubation periods, and inflammatory biomarker evaluation to further clarify the relationship between corrosion behavior, surface properties, and cytotoxicity under clinically relevant conditions.

## 5. Conclusions

Within the limitations of this in vitro study, commercially pure titanium (cpTi) demonstrated the most favorable biological response and exhibited no detectable cytotoxic effects on human gingival fibroblasts. In contrast, silver–palladium (Ag-Pd) and nickel–chromium (Ni-Cr) alloys demonstrated greater cytotoxicity, with Ni-Cr exhibiting the highest cytotoxic effect. Artificial saliva enhanced the cytotoxic response of all tested alloys compared with DMEM, indicating that both alloy composition and the surrounding environment influence the biocompatibility of dental metallic biomaterials. Future studies should quantify metallic ion release, evaluate multiple incubation periods, and investigate additional biological and inflammatory responses under conditions that more closely simulate the clinical oral environment.

## Figures and Tables

**Table 1:** Composition of casting alloys used in this study.

Alloy	Manufacturer	Trade Name	Alloy Composition/Description
Commercially Pure Titanium (cpTi)	Nippon Steel Corporation, Tokyo, Japan	ASTM Grade 2 cpTi	Bar, 10 cm length × 4 mm diameter
Silver-Palladium (Ag-Pd)	Ivoclar Vivadent, Schaan, Liechtenstein	IPS d.SIGN^®^ 53	Pd 53.8%, Ag 34.9%, Sn 7.7%, In 1.7%, Zn 1.2%, Pt < 1%, Re < 1%, Ru < 1%, Li < 1%; supplied as cylinders (6 mm diameter × 3 mm thickness)
Nickel-Chromium (Ni-Cr)	Ivoclar Vivadent, Schaan, Liechtenstein	IPS d.SIGN^®^ 15	Ni 58.7%, Cr 25.0%, Mo 12.1%, Si 1.7%, Fe 1.9%, Co < 1%, Ce < 1%; supplied as cylinders (30 mm length × 6 mm diameter)

**Table 2:** Cytotoxicity results of different alloys on HGF cells.

Alloy	cpTi	Ag-Pd	Ni-Cr	Control
DMEM Median Score Cytotoxicity	0.5	1.0	2.5	0.0
Artificial Saliva Median Score Cytotoxicity	0.5	1.5	3.0	0.0

Not cytotoxic = 0–0.5; Mildly cytotoxic = 0.6–1.9; Moderate cytotoxic = 2–3.9; Severe cytotoxic = 4–5.

**Table 3:** Viability (%) of HGF in response to different alloys in DMEM media and artificial saliva in comparison to negative control.

Viability %	Negative Control	cpTi	Ag-Pd	Ni-Cr
DMEM Media				
Range	90.7–92.3	88.7–90.8	75.8–79.4	65.4–69.3
Mean ± SD	91.8± 0.7	90.2± 0.51	77.6± 1.53 ^ab^	67.5± 1.54 ^abc^
Artificial Saliva				
Range	90.1–90.7	78.9–90.5	63.5–66.5	51.5–56.4
Mean ± SD	90.4 ± 0.22	86.3± 0.99	65.3 ± 1.14 ^ab^	54.1± 2.07 ^abc^

^a^ = comparison to negative control; ^b^ = cpTi verses other alloys; ^c^ = Ag-Pd verses Ni-Cr; *p* is significant at ≤0.05.

**Table 4:** Cytotoxicity (MTT) of elements released from different alloys in DMEM media and artificial saliva on HGF cells in comparison to negative control.

Cytotoxicity (MTT) Absorption at 492 nm	Negative Control	cpTi	Ag-Pd	Ni-Cr
DMEM Media				
Range	0.455–0.461	0.404–0.417	0.311–0.325	0.209–0.218
Mean ± SD	0.458 ± 0.002	0.411 ± 0.005	0.316 ± 0.006 ^ab^	0.213 ± 0.003 ^abc^
Artificial Saliva				
Range	0.401–0.409	0.379–0.388	0.291–0.297	0.201–0.207
Mean ± SD	0.405 ± 0.003	0.383 ± 0.003	0.294 ± 0.002 ^ab^	0.204 ± 0.003 ^abc^

^a^ = comparison to negative control; ^b^ = cpTi verses other alloys; ^c^ = Ag-Pd verses Ni-Cr; *p* is significant at ≤0.05.

## Data Availability

The original contributions presented in this study are included in the article. Further inquiries can be directed to the corresponding author.

## Abbreviations

The following abbreviations are used in this manuscript:

HGF	Human gingival fibroblasts.
cpTi	Commercially Pure Titanium
Ag-Pd	Silver-Palladium
Ni-Cr	Nickel-Chromium
